# Trauma systems in high socioeconomic index countries in 2050

**DOI:** 10.1186/s13054-024-04863-w

**Published:** 2024-03-16

**Authors:** Tobias Gauss, Mariska de Jongh, Marc Maegele, Elaine Cole, Pierre Bouzat

**Affiliations:** 1 Division Anesthesia and Critical Care, University Hospital Grenoble Alpes, Grenoble, France; 2 Grenoble Institute for Neurosciences, Inserm, U1216, Grenoble Alpes University, Grenoble, France; 3 Network Emergency Care Brabant (NAZB), ETZ Hospital, Tilburg, The Netherlands; 4 Department of Traumatology and Orthopedic Surgery, Cologne-Merheim Medical Center, University Witten-Herdecke, Cologne, Germany; 5 Centre for Trauma Sciences, Blizard Institute, Queen Mary University of London, London, UK

**Keywords:** Trauma systems, Epidemiology, Disaster preparedness, Education, Governance, Funding, Prediction

## Abstract

Considerable political, structural, environmental and epidemiological change will affect high socioeconomic index (SDI) countries over the next 25 years. These changes will impact healthcare provision and consequently trauma systems. This review attempts to anticipate the potential impact on trauma systems and how they could adapt to meet the changing priorities. The first section describes possible epidemiological trajectories. A second section exposes existing governance and funding challenges, how these can be met, and the need to incorporate data and information science into a learning and adaptive trauma system. The last section suggests an international harmonization of trauma education to improve care standards, optimize immediate and long-term patient needs and enhance disaster preparedness and crisis resilience. By demonstrating their capacity for adaptation, trauma systems can play a leading role in the transformation of care systems to tackle future health challenges.

## Background

Despite improvements in prevention and clinical management, injuries remain a leading cause of death [1, 2] and a significant public health concern in countries with a high socioeconomic development index (SDI). According to the 2019 Global Burden of Disease Initiative mechanisms including falls, road traffic incidents and interpersonal violence accounted for 8% of Years Lived with Disability (YLD) across all age groups, compared to less than 1% for ischemic heart disease, less than 2% for ischemic stroke and 2% for all neoplasms [1]. This burden of disease in high SDI countries remained stable since 1990 in terms of YLD (around 8%) and deaths (around 7%) [1]. In response to this public health concern, several high SDI countries, such as the UK, Germany, the Netherlands, parts of North America and Australasia, have introduced organized trauma systems [2–9]*.*

Trauma systems offer a comprehensive framework for complex healthcare issues yet remain contingent on their socioeconomic context. Over the next 25 years, this context will experience considerable transformation such as changing epidemiology, contested healthcare expenditures and the challenge to maintain a competent clinical workforce [10–12]. At the same time, therapeutic and rehabilitative strategies will advance, and as more people survive traumatic injury there will be an even greater emphasis on recovery and functional outcome.

Considering the operational and structural role of trauma systems, and their ability to respond to predicted and unplanned crises, a review of future challenges and how trauma systems could adapt is warranted. The objective of this review is to raise awareness and stimulate a constructive debate to plan trauma systems for the next two to three decades. Changes in epidemiology, the structure of trauma systems, governance, funding, learning systems and trauma education will be discussed. Given the lack of robust evidence, risk of bias and uncertainty on future political trajectories, this review assumes that the current existing societal and economic frameworks in high SDI countries, without major conflict or internal upheaval, will remain comparable.

### Trauma systems in high SDI countries

Over the past four decades, comprehensive systems of care, *Trauma Systems*, have evolved with the specific remit of identifying and managing major trauma to reduce mortality and morbidity in trauma [6, 13–15]. These systems integrate all aspects of management from injury prevention, triage, pre-hospital care, patient transfer, initial resuscitation, definitive care to rehabilitation and from governance and funding to quality control. Trauma systems in high SDI countries reduce mortality [16] and improve functional outcome [17, 18]. Exclusive trauma systems send patients to a small number of specifically designated centers, thereby centralizing expertise [19, 20]. Inclusive trauma systems within defined geographical regions designate trauma receiving hospitals according to resource availability, to match patient needs to those resources [3, 16, 21] and care for the most severely patients in designated centers according to a *hub and spoke* model [3, 7, 8, 22]. Beyond clinical provision, a mature trauma system has responsibility for injury prevention, data and governance, education, research and mass casualty preparedness and response, all of which can challenge organizational structures and financial constraints even within high SDI countries [23]. The beneficial effects conferred by mature trauma systems are consistent [16, 18, 24]; however, the optimal future organizational model is yet to be universally agreed or adopted [5, 22, 25, 26]. A multitude of governance and financing structures exist across and coexist within high SDI countries [16, 19, 27]. Table 1 illustrates three governance prototypes: a *trauma-community model, state agency driven model*, *non-state driven agency model*.

**Table 1:** Governance prototypes of trauma systems: trauma-community model, state agency model, non-state agency model

	Trauma-community model	State agency model	Non-state agency model
Driver of process	Trauma community (professionals, organizations)	State agency (Ministry, Health Agency)	Non-state actor (Medical/Scientific society)
Lead agency	Not on national level heterogenous (regional, none)	Legal authority to designate and certify centers	No legal authority, strong normative power
Networks based on population need	Not on national level heterogenous (regional, none)	Yes	Yes
Formal process for center designation	Not on national level heterogenous (regional, none)	Yes	Yes
Independent assessment	Not on national level heterogenous (regional, none)	Yes	Yes
Information system (registry)	Heterogenous	Yes	Yes
System monitoring	Heterogenous	Yes	Yes
Education standards	Heterogenous	Yes	Yes
Funding	Heterogenous	Yes	Yes (non-state)
Triage guidelines and SOP	Heterogenous	Yes	Yes (non-state)
Examples	USA, France, Spain, Italy	Netherlands, UK, Norway	Germany

### Which external trajectories are likely to change trauma epidemiology?

Among the numerous external trajectories and demographic shifts in high SDI countries that might affect trauma epidemiology over the next 25 years, the effects of an aging population, evolution of transportation, self-harm and interpersonal violence are major contributors.

#### Aging population

It is expected that by 2050 20% of high SDI populations will be aged 70 years and older, compared to the current rate of 14% [28]. Several systems report an increasing shift in the median age of trauma patients [29, 30] and low energy mechanisms such as falls are predominating and will continue to increase [31]. Current population level falls that prevent programs are effective, comprising awareness, muscle and proprioceptive training and environmental hazard control, yet are workforce intensive [32–34]. Scientific and technological innovation is required to improve on this to meet the needs of aging populations.

While the proportion of elderly will increase, the patterns of aging and roles will evolve [35, 36]. Many older people will pursue dynamic social, economic and cultural roles, and increased physical activity. This physical activity will maintain and improve their physiological and cognitive reserve in response to injury yet at the same time increase their exposure to risk and more high energy trauma mechanisms such as sports, cycling and motorbikes. These changes mandate an individualized approach to elderly trauma patient where triage and assessment focus on *physiological age* and *frailty* rather than *chronological age* [37, 38]. Sensitive triage will facilitate pathways for non-frail, healthy elderly patients with good physiological reserve to high-level centers for aggressive management by specialized teams. The existing Geriatric Trauma Outcome Score does not account for frailty and preexisting levels of autonomy [39] despite recent evidence highlighting the need for robust, valid, geriatric trauma triage tools which incorporate physiological aging and frailty [40, 41]. Geriatric trauma pathways integrating decision support tools for prognostication, palliation and patient choice should also be part of every trauma program. Dedicated multidisciplinary management and early rehabilitation teams improve outcome in trauma patients [42]. The trauma community will need to decrease the aging-related knowledge gap and intensify research into triage, management, prognostication and rehabilitation in the older trauma patient. Considerable agency leadership and institutional efforts will be required to make this transition happen.

#### Evolution of transportation

Deaths from transportation in high SDI countries decreased from 20 to 11/per 100,000 over the last 30 years [1]. Improved vehicle safety design has reduced mortality in crashes over the last 20 years [24, 43, 44]. Future prevention strategies, including automatic breaks, speed and collision control, automatic vehicle deactivation triggered by sleep or drug-induced driving patterns, may improve safety further. Self-driving cars are expected to reduce road traffic accidents, although not supported by current evidence; and incidents with self-driving vehicles may increase before the technology reaches maturity [45]. Injuries from electric scooters comparable to those seen with motorbikes illustrate the difficulty to predict the epidemiological effects of new technology [46–49]. The prevention of transportation incidents is not only restricted to new technologies. Despite a comparable SDI, the USA accounts for more than twice the mortality rate of pedestrians than Australia, namely 12.5 versus 5.3 deaths/100,000 per year [50]. Pedestrian deaths in the USA increased for the first time in 41 years according to the State Highway Safety Office in 2022 [51], demonstrating considerable uncertainty in our understanding of how road safety evolves, to provide projections and determine the most efficient prevention policies [52]. Programs with a consistent beneficial impact adopted proactive system approaches, where human behavior is considered an integral dimension to improve traffic safety [52]. Prospective monitoring of emerging trends in transportation injuries shared by researchers, epidemiologists and policymakers will be crucial to assess the effect and efficacy of preventive policies and enhance adjustment to risk patterns. Trauma systems, transportation industries, lead agencies and policy makers must intensify coordination and cooperation in future years and decades.

#### Self-harm and Interpersonal violence

Injuries caused by self-harm have had a stable incidence of 15 deaths/10,000 per year across high SDI countries since 1990, with some countries even reporting significant reductions, such as Finland by 50% [1]. Similar to violence, circumstances with social and economic instability, including natural disaster, increase the risk of suicide [53–56], and in the future, the risk should be reduced by prioritizing social stability and providing equitable access to mental health.

**Fig. 1:**
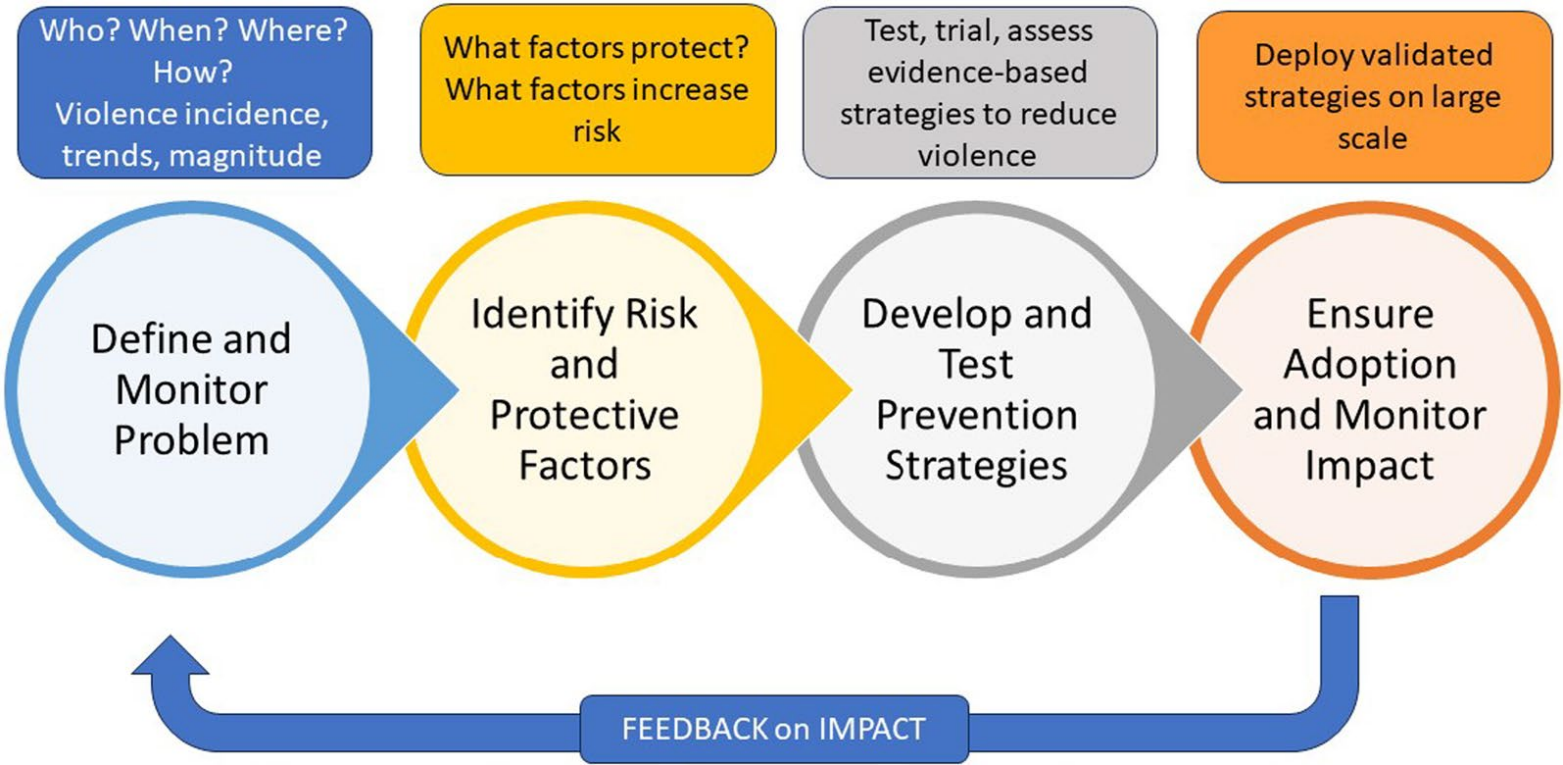
Public Health Approach to interpersonal violence, adapted from Dahlberg et al. [62]

In high SDI countries, deaths from interpersonal violence decreased from 4.2 to 2.5 deaths/10,000 per year [1]. Yet despite this small decrease in absolute numbers, interpersonal violence generates substantial societal and psychological impacts. Patterns of violence are similar across most high SDI countries, except for mass shootings (defined as > 4 fatalities per incident) in the USA, with an increase from 272 in 2014 to 651 in 2023 [57]. The sociology and economics of violence are well understood [58, 59], where social instability, limited access to mental health support, availability of drugs/alcohol, economic deprivation, gender and social inequalities are among the numerous risk factors. There are, however, considerable discrepancies within and among countries, where access to firearms increases the risk of death and injury at population level, such as the 7.5 times higher homicide rate in the USA than in comparable high SDI settings [60]. The increased domestic assaults reported during the COVID-19 pandemic also illustrates the potential for future increases in interpersonal violence [61]. The most promising avenue for violence prevention is described within a *Public Health Approach* [62] (Fig. 1). This approach requires intensive multilayer cooperation from the community (anti-violence training, employment, education, policing) to legislative bodies (access to weapons, social rehabilitation, poverty prevention) [63]. Advocacy and monitoring through healthcare providers such as Hospital Based Violence Intervention Programs reduce violence (https://www.thehavi.org/what-is-an-hvip); and numerous public health initiatives report dramatic reductions [64]. An important consideration remains the long-term support of victims of violence, with need for improvement in many countries according to the WHO [65].

#### Alternative trajectories

Apart from the aforementioned factors, migration and climate change, civil disorder and armed conflict will probably affect how we provide trauma care in high SDI. The trajectories associated with these factors are difficult to predict and their impact may destabilize or destroy the current socioeconomic framework of high SDI countries [66–68]. The trauma community should prepare for these scenarios and learn how to make our trauma systems resilient in a challenged or failing socioeconomic environment. Some examples of political turbulence have generated high levels of civil unrest while the overall social and economic order remains intact. For example, during the mass terrorist events witnessed, existing trauma provision appeared to cope with the scale of unrest. A recent international follow-up survey conducted in 22 hospitals exposed to mass attacks identified the many elements of crucial lessons learned, even years after the event. These included the need for re-triage at hospital arrival, need for coordination roles, flexibility and large-scale training exercises. The organization of human response, rather than consumption of physical supplies, emerged as the key finding [69], and this emphasizes the need to prioritize this during training now and in the future. Innovative tools for mass casualty management have been developed by an international consortium [70], yet these innovations rely on high levels of technicality, creating dependency and increasing the risk of reduced resilience and adaptation. A multi-casualty crisis is first and foremost a cognitive challenge and large-scale training, including civilians, appears to offer the most cost-efficient preparation. The Ukraine conflict may provide useful lessons on how to prepare a civil society for crisis and recruit civilians into crisis management roles [71] with a possible first lesson on tourniquet use [72]. In the future, this type of preparation could be combined with the training and deployment of highly skilled medical teams able to provide advanced trauma care even in hostile civil environments, comparable to the experience of US and UK forces [73].

## Governance challenges and perspectives

Despite their differences, trauma systems face similar challenges. One challenge concerns the balance between central regulation and compliance to ensure that hospitals or networks meet standards and guidelines, while allowing sufficient operational autonomy [2, 8]. Compliance can either be obtained with sanctions or incentives (best practice tariff UK [74]) where a lead agency is required to allocate or restrict resources or authorizations. Inclusive systems enable competence in lower-level centers and are possibly more patient centered being closer to families and community care, which may enhance psychological comfort, social integration and reduce indirect cost for families and lost productivity [2].

**Fig. 2:**
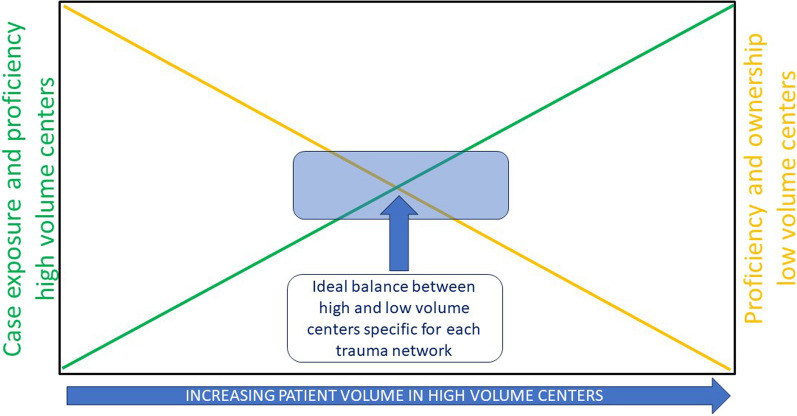
Theoretical Volume-Proficiency-Ownership relationship in trauma networks; the higher the volume in referral centers (= level-1 centers), the higher their level of case exposure and proficiency, the higher the risks of decreased proficiency and network ownership of lower volume (= level-2 and 3); (adapted from Hietbrink et al. [76]

Centralized trauma systems run the risk of deskilling clinicians and staff in peripheral hospitals, reducing their sense of ownership and commitment to their trauma network. Reduced exposure to critically injured trauma patients may exacerbate fragile competence levels and psychological apprehension and increases the need for evidence-based guidelines to reduce the threshold for referral to high-volume centers [75]. This reduced exposure may decrease survival in critically injured patients unfit for evacuation to higher-level care. Yet, management of critically injured patients in high-volume centers is consistently associated with better outcomes across different trauma systems [9, 75, 76]. The appropriate volume threshold depends on each trauma system and network. German and Dutch networks obtain comparable outcomes with lower volumes compared to US networks, and there may be a theoretical balance between volume, proficiency and ownership for every network between high and low volume centers (Fig. 2). Proactive outcome monitoring across networks, multimodal training [77] and telemedicine [78] for lower-level centers are pragmatic responses and require time and human resources and transparent data sharing.

Another challenge concerns the trauma workforce although the issue is not restricted to trauma care. The evolution of the clinical work force impacts trauma education and outcome [79, 80]. High staff turnover, decreasing staff retention, increasing locum work and the evolving self-perception of healthcare professionals and their relationship to work result training exercises and skill maintenance being taxing for some [81–83]. Increasing levels of sub-specialization exposes peripheral hospitals to a frequent absence of a trauma-experienced work force [16]. There appears to be no panacea for improving staff retention and reducing turnover other than education, clear career pathways, manageable workload, specific financial individual and institutional incentives. Yet going forward staff retention and protection may be improved if trauma systems actively and preemptively acknowledge the psychosocial burden on trauma professionals by being consistently exposed to disturbing events. A recent review from the Royal College of Emergency Physicians identifies lack of support, leadership and a culture of blame and negativity as barriers to staff retention [84] and issued a Psychologically Informed Practice Policy (https://rcem.ac.uk/psychologically-informed-practice-and-policy-pipp/). Several of the recommended interventions are associated with improved stress resilience [85] and prevent [86] or attenuate post-traumatic stress disorder [87]. Table 2 summarizes governance challenges and solutions (Table 2).

**Table 2:** Governance challenges, perspectives, solutions and examples

	Governance		
	Challenge	Perspective solution	Example
Quality control	Ensure safety and guideline compliance	Automatic feedback on process and clinical KPI Local and central audit	KPI Dashboard shared across network [8] Rating standardized mortality [8]
Compliance incentive	Reward or sanction performance and compliance Reward positive initiative	Structural, financial incentives Performance feedback to providers, local audit	Best Practice Tariff in UK, incentivized for MTC [74] Network event and tracking system [102]
Centralization versus subsidiarity	Balance between lead agency control and center autonomy	Sufficient resource allocation and competence to preserve subsidiarity	DGU Trauma Netzwerk, UK, Norway, Netherlands [12, 15]
Patient volume	Balance between center volume and exposure and skill level	Quality and KPI control, education	USA, Netherlands, Germany, UK, Norway, Australia, Canada [12, 15, 21, 24, 31]
Patient involvement	Keep care and process patient centered	Associate patients and NOK to governance, audits, priority setting	UK, Canada, Australia [8, 15, 31, 37, 103–105]
Rehabilitation capacity and pathway	Insufficient rehabilitation capacities	Calibrate rehabilitation capacity on patient volume Patient-centered trajectories	Australia [37, 95, 103–105]

KPI, Key Performance Indicator; MTC, Major Trauma Center; DGU, Deutsche Gesellschaft für Unfallchirurgie

Trauma systems will require additional roles such as system administrators, data scientists/statisticians, informatic engineers, psychologists and risk engineers. These roles are essential to operate an adaptive, responsive trauma system [3, 5, 18]. Information systems increase the capacity to exploit the intelligence available to inform decision making and facilitate system governance and oversight [88, 89]. Future trauma systems need to acquire these structural roles and skills, while recognizing that some, including clinical roles, may disappear or evolve due to technological progress.

## Funding challenges and perspectives

Despite the high socioeconomic impact and disease burden, trauma care remains underfunded. In 2015 in the USA, an estimated 11.8% gap persisted between total funding and disease burden measured by DALY (Disability-adjusted Life Years), compared to cancer and infectious disease [25]. Comparable data from the Netherlands indicate disparate levels of funding according to age, gender and injury type [90]. Available evidence suggests a rural to urban and academic to non-academic funding gradient, where state funded systems with mandatory national insurance receive more reliable funding [15, 18, 25, 26]. Trauma care and networks are resource intense and rarely profitable, and the actual costs incurred are infrequently compensated in full and many trauma centers work at a loss. Diagnosis-related tariffs tend to underestimate the complex cost associated with a trauma case and compare unfavorably to other pathologies [91]. Some systems attempt to compensate by trauma activation fees to reimburse the high resource intensity trauma requires [92]. A comprehensive analysis of cost in the New Zealand demonstrates a cost reduction per DALY in a mature trauma system [93]. Going forward, the trauma community should learn from other industries and NGOs to accelerate technology transfer and develop public–private partnerships. Such initiative should have the capacity to generate alternative and feedback funding and reimburse health data use and medical expertise to fund trauma systems and develop innovative funding schemes. Private–public partnerships can take the form of human resource patronship, where private partners do not pay directly for collaborators, but provide time and expertise to trauma systems. Future healthcare funding will become more constrained than under current circumstances. As a clinical and scientific community, we may need to prepare for the scenario where socioeconomic scenarios change to a point where provision of complex care and novel innovation is extremely challenged (Table 3).

**Table 3:** Funding challenges, perspectives, solutions and examples

	Funding		
	Challenge	Perspective solution	Example
Compensate resource intensity trauma	Compensate for trauma care capacity and readiness	Trauma Readiness Fee	USA [90, 93]
Dependency on central funding by lead agency	Insufficient budget or resource autonomy at center and network level from central funding and lead agency	Develop alternative funding sources	USA: fees from fine, fees vehicle registration and insurance [90] Charity, lottery (UK HEMS)
Technology development and knowledge transfer	Technology pipeline not matched to clinical needs Insufficient compensation of knowledge transfer from healthcare providers to industry	Public–Private partnership, joint labs Channel percentage of industry revenues to healthcare organizations	Technology transfer desk, conceive partnership from start of scientific pipeline Incorporate revenue models and provide legal framework, account for corporate responsibility (automobile, sport,…)
Inadequate staffing and material resources	Inflexible staff recruitment and incentive mechanism Inflexible and long public provision process for material	Decentralize recruitment and incentive mechanism Simplify pipeline for provision	Competitive recruitment of health professionals and experts (psychologist, data and computer science, network specialists)

HEMS, Helicopter Emergency Service

## A learning trauma system

Knowledge empowerment for trauma providers at all levels remains a leverage for adaptation and resilience. To adapt and learn the trauma community requires stronger ties with the civil society, patients and their next of kin, policy stakeholders, NGOs and industry partners. These ties are necessary to identify needs and trends, anchor trauma care in e society and shape a partnership to incorporate patient preference and priorities into future trauma systems [94, 95] such as the long-term patient-centered outcome of the PATCH-trial [96]. The trauma community could learn from the example of advocacy for other pathologies [97, 98] and one example is represented by the Coalition for National Trauma Research (nattrauma.org). Advocacy means that we need to acknowledge the risk of trauma in the life trajectory of many vulnerable groups (self-harm, alcohol and drug dependencies, high-risk behavior, violence).

**Fig. 3:**
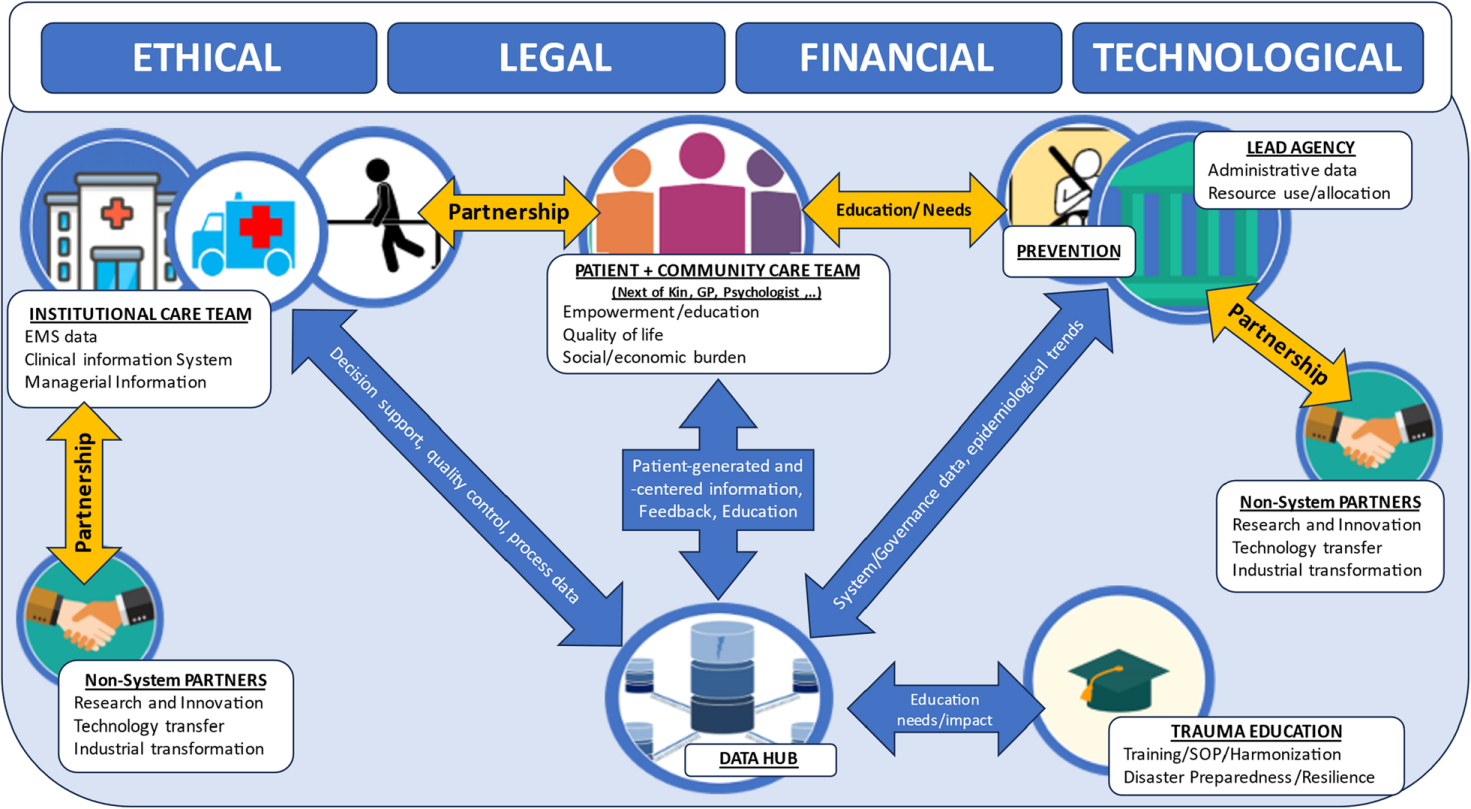
Structure of a learning trauma system; each blue oval represents specific building blocks of the framework (ethical, legal, financial and technological) required as prerequisite for the learning trauma system to be implemented and allow comprehensive, safe and shared information use

Measurement against Key Performance Indicators constitutes a quintessential component of trauma systems [99]. Most systems rely on labor- and resource intense centralized registries with a high human workload and restriction of real-time data exploitation [100]. Despite abundant collection, information remains fragmented into provider organizations and data silos that restrict exploitation [101, 102]. The evolution of data sciences enables collection of large multi-scale and disease-related data into dedicated data hubs [103]. These hubs can collect centralized or decentralized aggregated information. Automatic retrieval and federated learning will create decentralized *learning systems* to provide KPIs for governance and real-time decision support [104]. Such system allows to detect trends, follow up on long-term patient-centered outcomes to learn about patient preferences and incorporate their priorities into research and policy [105–108]. This scenario should also recognize the technical, structural legal and conceptual challenges that lie ahead before a comprehensive deployment is feasible. The protection of patient and patient and health provider interest should be enshrined into a dedicated legal, ethical, financial and technological framework (Fig. 3) essential to develop [102, 103]. The legal and ethical aspects and the safe transfer of these technologies and necessary knowledge constitute the core of this challenge; however, the detailed discussion of is beyond the scope of this review.

## Trauma education

The pioneering deployment of the Advanced Trauma Life Support course (ATLS) demonstrated the life-saving benefit of a structured and standardized approach to trauma [109]. The trauma literature provides a strong signal for the association of standardized trauma education and improved patient outcome [110]. Yet, trauma education remains context dependent across high SDI countries with considerable regional and international variability regarding doctrine, standards and assessment [111, 112]. The line between doctrine and dogma is thin. Several dogmas persist within the trauma community, such as the Golden Hour, permissive hypotension, all shock is bleeding, and vasopressors are harmful. At the same time trauma management is subject to constant change. Plasma and blood appear as substitute for fluid, intubation rates fall and supraglottic devices considered a serious alternative. To drive future system changes and convert dogma to doctrine, there seems to be no alternative to a rigorous scientific process.

To date there has been a focus on medical professionals providing trauma care [26, 113–115] while neglecting the proficiency and experience of the clinician [116], or the fact that several systems have successfully empowered highly proficient non-physician providers [3, 22, 25, 99, 111]. Patients require an interdisciplinary and multi-professional trauma team with different roles, and skillsets, some are shared and overlapping and some exclusive [4, 5, 15, 18, 21]. Future trauma education should acknowledge the complementary contribution of various roles and skillsets to encompass the entire patient pathway from scene to rehabilitation [99, 117]. These education pathways could define mandatory technical and non-technical skill sets for each role within the trauma pathway rather designate specific subspecialties and professions [118–121]. According to regional or national context, in some systems paramedics will acquire specific skills sets while in other systems they will be restricted to physicians. What matters is the standardized and tested proficiency and competence. Nevertheless, some skills are too complex to be deployed at system level and can only be safely offered by a restricted set of highly trained providers (REBOA, thoracotomy, ECMO, decompressive craniectomy, neuro-ICU) [122–125]. Scientific societies should work toward an international standardization and harmonization of evidence-based training curricula and assessment. These curricula require systematic disaster preparedness and resilience training to enhance the cooperation between civil, rescue, law enforcement and the military. Trauma systems have and will continue to play a fundamental role in the response to crisis and disaster [69, 126] including the SARS-Cov-2 pandemic [127], and several organizations and countries lead by example [23, 128–131].

Mandatory training and assessment relies on strong institutional commitment [112]. Across all trauma systems in high SDI countries, time is a precious resource; the pressure to “produce” care restricts the time available for training and education. In comparison with other high-risk industries, healthcare assigns insufficient amounts of mandatory time for education of entire teams despite proven benefit [110, 118]. Trauma care organizations will need to increase the active workforce to account for the time to facilitate mandatory team training. Work force evolution and staff turnover require complementary, accessible methods such as eLearning and virtual reality, as suggested by pilot studies [132–134]. These technologies are available on-demand and can facilitate repetition and habituation but can be no substitute for traditional training or in situ team training, as they lack the immediacy and authenticity of interpersonal interaction and communication. In an analogy with bystander cardiopulmonary resuscitation, there is currently a knowledge gap of how to better integrate civilians into the trauma pathway, how to train them in basic trauma life support, tourniquet use or intramuscular tranexamic acid administration [135–137], and this requires future consideration and consolidation.

## Conclusion

Societies in high SDI countries will experience profound structural and socioeconomic change over the next two to three decades. These changes will reverberate on the epidemiological patterns, needs and threats to existing healthcare systems. Few pathologies other than trauma involve such a large array of professions and roles in the healthcare system. Therefore, trauma care exerts a comprehensive influence on entire healthcare systems with a considerable potential for transformation. Dealing with uncertainty, adaptation and resilience are quintessential elements of the *trauma mindset*. Hence, trauma systems should take a lead to learn and evolve in governance, funding, education and information science to help face the complex challenges of the next 25 years.

## Data Availability

Not applicable.

## Abbreviations

ASC-COT: American College of Surgeons Committee on Trauma
AIS: Abbreviated Injury Severity
DALY: Disability-adjusted Life Years
ICU: Intensive care unit
IM: Intramuscular
ISS: Injury Severity Scale
KPI: Key Performance Indicator
NGO: Nongovernmental organization
PREHOSP: Prehospital
REBOA: Resuscitative endovascular balloon occlusion of the aorta
SDI: Socioeconomic index
TXA: Tranexamic acid
UK: United Kingdom
WHO: World Health Organisation American College of Surgeons Committee on Trauma
